# Comparative Phenotypic and Transcriptomic Analysis Reveals Key Responses of Upland Cotton to Salinity Stress During Postgermination

**DOI:** 10.3389/fpls.2021.639104

**Published:** 2021-04-13

**Authors:** Jingxia Zhang, Pei Zhang, Xuehan Huo, Yang Gao, Yu Chen, Zhangqiang Song, Furong Wang, Jun Zhang

**Affiliations:** ^1^Key Laboratory of Cotton Breeding and Cultivation in Huang-Huai-Hai Plain, Ministry of Agriculture, Cotton Research Center of Shandong Academy of Agricultural Sciences, Jinan, China; ^2^Key Laboratory of Plant Stress Research, College of Life Sciences, Shandong Normal University, Jinan, China

**Keywords:** salinity tolerance, root, germination, transcriptome, signal transduction pathways, phytohormone, ethylene transcription factor 12

## Abstract

To understand the molecular mechanisms of salinity tolerance during seed germination and post-germination stages, this study characterized phenotypic and transcriptome responses of two cotton cultivars during salinity stress. The two cultivars were salt-tolerant (ST) LMY37 and salt-sensitive (SS) ZM12, with the former exhibiting higher germination rate, growth, and primary-root fresh weight under salinity stress. Transcriptomic comparison revealed that up-regulation of differentially expressed genes (DEGs) was the main characteristic of transcriptional regulation in ST, while SS DEGs were mainly down-regulated. GO and KEGG analyses uncovered both common and specific responses in ST and SS. Common processes, such as reactive oxygen species (ROS) metabolism and cell wall biosynthesis, may be general responses to salinity in cotton. In contrast, DEGs involved in MAPK-signaling pathway activated by ROS, carotenoid biosynthesis pathway and cysteine and methionine metabolism pathway [producing the precursors of stress hormone abscisic acid (ABA) and ethylene (ET), respectively] as well as stress tolerance related transcription factor genes, showed significant expression differences between ST and SS. These differences might be the molecular basis leading to contrasting salinity tolerance. Silencing of *GhERF12*, an ethylene response factor gene, caused higher salinity sensitivity and increased ROS accumulation after salinity stress. In addition, peroxidase (POD) and superoxide dismutase (SOD) activity obviously declined after silencing *GhERF12*. These results suggest that *GhERF12* is involved in salinity tolerance during early development. This study provides a novel and comprehensive perspective to understand key mechanisms of salinity tolerance and explores candidate genes that may be useful in developing stress-tolerant crops through biotechnology.

## Introduction

Salinity is one of the primary abiotic stresses that adversely affect global crop production. Multiple growth inhibitions due to salinity usually cover the entire growth period (Panta et al., 2014). It was estimated that more than 6% of the world’s 800 million agricultural lands was affected by high salinity (Setia et al., 2013). Owing to natural reasons and agricultural practices such as irrigation, the proportion of salinity-affected agricultural land is increasing annually (Zorb et al., 2019), heightening the urgency in demand for developing salt-tolerant crop varieties. The development of tolerant crops will be facilitated by uncovering tolerant genes and underlying molecular mechanisms.

Salinity causes water deficits, ion stress, inhibitions of essential enzymes, decreases in water and nutrient supply, as well as higher levels of reactive oxygen species (ROS). These changes can cause membrane damage, protein oxidation, and DNA lesions, leading to irreparable metabolic dysfunction and even cell death (Miller et al., 2010; Noctor et al., 2014). Therefore, ensuring safer ROS levels is essential to plant cells under stress. Plants have non-enzymatic and enzymatic antioxidative systems to remove excess ROS. The former system includes carotenoids, ascorbate, reduced glutathione (GSH), and flavonoids; while the latter one including superoxide dismutase (SOD), peroxidase (POD), catalase (CAT), ascorbate peroxidase (APX), and glutathione reductase (GR) (Chen and Yang, 2019). Although toxic at high concentrations, ROS are also a signal molecule during plant growth, development, and stress responses (Baxter et al., 2014; Willems et al., 2016). It is not fully understood how the ROS signal is perceived up to now. However, a mitogen-activated protein kinase (MAPK) cascade and downstream transcription factors (TFs) seem to be key regulatory components of ROS signaling and responses. Activation of the MAPK signaling pathway regulates numerous TF genes that mediate the expression of ROS-generating and -scavenging enzymes. Some TF genes, including members of the WRKY, bZIP, MYB, HSF, and AP2/ERF families, have been shown to response to H_2_O_2_ stress. Additionally, other studies show that TFs play important roles in ROS signaling, either directly or indirectly. For instance, salt-responsive ERF1 in rice amplifies the ROS-activated MAPK cascade and translates the signal into an appropriate expressional response resulting salt tolerance (Schmidt et al., 2013).

Phytohormones are important regulators of plant development and stress tolerance. Abscisic acid (ABA) and ethylene (ET) are considered to be ‘stress hormone’ for their key roles in response to biotic and abiotic stresses. Numerous studies have shown that key genes linked to hormone biosynthesis and signal transduction are involved in salinity tolerance. For instance, *acs7* mutant, a T-DNA insertion in 1-aminocyclopropane-1-carboxylic acid synthase (ACS) which was the rate-limiting enzyme in ET biosynthesis pathway, showed diminished germination rate under salinity conditions relative to the control. Both ABA and ET possess their own biosynthesis and signaling pathways, but regulatory components in these biological processes generate complex and overlapping responses (Van den Broeck et al., 2017). Recent studies suggested that the ABA signaling TF, VViABF2, is involved in ET signaling. Moreover, ET response factor (ERF) SIPti4 is involved in seed germination and responses to drought stress through adjustments to ABA metabolism and signaling (Hopper et al., 2016; Sun et al., 2018).

Cotton is a leading natural-fiber and oil crop worldwide, as well as a pioneer crop for saline-alkali land utilization because of its moderate salinity tolerance. However, high salinity still negatively affects cotton growth and development during all developmental stages. Germination and post-germination are pivotal early-developmental periods for determining whether a seed can grow into a plant; meanwhile, the same stages are extremely sensitive to environmental stress, including high salinity. Numerous studies have explored salinity tolerant genes and the underlying molecular mechanisms in leaves and roots of adult cotton (Peng et al., 2014, 2018; Guo et al., 2015; Xu et al., 2020). However, studies on salinity tolerance during early development stage remain relatively scarce.

In the present study, two cultivars exhibiting contrasting salinity tolerance, salt-tolerant LMY37, (termed ST) and salt-sensitive ZM12 (termed SS), were used for phenotypic and transcriptome analysis during germination and post-germination. This study identified novel candidate genes for breeding salinity-tolerant cotton and provides comprehensive insight into the molecular mechanism of salinity tolerance during early developmental stages in cotton.

## Materials and Methods

### Two-Year Assay in Filed Condition and Laboratory Analysis for Salinity Tolerance

An assay of salinity tolerance in field conditions was performed in Binzhou City (soil salinity content: 0.45–0.55%, w/v) of China’s Shandong province during 2017 (April 30–October 30) and 2018 (May 01–October 28). The salinity tolerance of >600 cotton cultivars and germplasm was determined via measuring germination rate, survival rate, plant height, and fiber yield. Results from field data identified 12 cultivars with contrasting salinity tolerance, which were used for detailed tolerance analysis in a greenhouse. Laboratory experiments were conducted at the Sub-center of National Cotton Improvement at the Shandong Academy of Agricultural Sciences, Jinan City, Shandong, China.

Cotton seeds were surface-sterilized, planted in uniform pots containing nutrient soil with or without 100 mM NaCl, and left in the culture chamber for ∼15 days (28°C, 14/10 h light/dark). Germination and cotyledon unfolding rates were scored for 1–15 days. Seeds that emerged from the soil were considered germinated. Primary root length was photographed and measured using Image J^1^.

### RNA Isolation and Sequencing

Seeds of LMY37 (ST) and ZM12 (SS) were surface-sterilized and soaked overnight at 28°C. The seed coat was completely removed and naked seeds were covered with sterilized vermiculite until the primary root length was 1.0–1.5 cm. Plantlets were then exposed to 100 mM NaCl or mock conditions (0 mM NaCl) for 24 h. Roots of 15 independent ST and SS plants were then collected to make three biological replicates for RNA-seq. Preparation and sequencing of 12 mRNA libraries on HiSeq (Illumina) were out-sourced to Novogene (Beijing, China). Adapter, poly-*N*, and low-quality reads were removed from raw data for subsequent analysis. In addition, Q20, Q30, and GC content of these cleaned reads were calculated. The data presented in the study are deposited in the NCBI SRA BioProject repository, and the accession number is PRJNA684671.

Differential expression analysis was performed using the DESeq 2 R package. The *P*-values were adjusted using Benjamini and Hochberg’s approach for controlling the false discovery rate. Only genes with an adjusted *P* (padj) < 0.05 were considered differentially expressed genes (DEGs). Gene ontology (GO) enrichment analysis of DEGs was implemented with the clusterProfiler R package, in which gene-length bias was corrected. GO terms with padj < 0.05 were considered significantly enriched. Enriched pathways were determined using the Kyoto encyclopedia of genes and genomes (KEGG)^2^, and clusterProfiler was again used to determine significantly enriched KEGG pathways.

### Virus-Induced Gene Silencing in Cotton and Salinity Tolerance Analysis

Gene-silenced plants were constructed using seed-soak agroinoculation (SSA)–VIGS as previously described (Zhang et al., 2018), with some modifications. Plump cotton seeds were surface-sterilized with 3% hydrogen and then immersed in sterile dd H_2_O for 6 h at 28°C. Seed coats were completely removed and naked seeds were dipped into *Agrobacterium* suspensions (OD 600 = 1.5 for 8 h), then incubated in the dark for 24 h. Inoculated seeds were directly planted into sterile vermiculite (26°C for 3 days in darkness) and then moved into the culture chamber (26°C, 14 h light/10 h dark).

Approximately 8–10 days after inoculation, roots of *GhERF12*-silenced and vector-control plants were harvested for qRT-PCR to detect gene silencing. And then, equal numbers of silenced (at least 10 seedlings) and vector control (at least 10 seedlings) plants were exposed to 100 or 0 mM within an appointed time period for salinity tolerance analysis. Staining with DAB and trypan blue were used to determine ROS accumulation and cell death, following published methods (Thordal-Christensen et al., 1997; Gao et al., 2013). Enzyme activities of superoxide dismutase (SOD) and POD were calculated as described by Wang et al. (2015). Experiments were performed in triplicate.

### qRT-PCR

Total RNA was extracted from roots using TRIzol reagent (Invitrogen)^3^ following manufacturer protocol. Complementary DNA was synthesized using a PrimeScript RT reagent kit with gDNA eraser (TaKaRa). Cotton *Actin9* (*GhActin9*) was selected for normalization. Primers were designed in Primer Premier 5.0 (Premier Biosoft International, Palo Alto, CA, United States). Each 50 μL reaction sample was run on a Bio-Rad IQ2 sequence detection system with Applied Biosystems software. Relative expression was calculated using the 2^−ΔΔCt^ method.

### Data Analysis

Data were analyzed by SPSS 17.0 (SPSS, Chicago, IL, United States) and presented as means with SD. The data significance statistical analysis was determined by Student’s *t*-test at significance level of *P* < 0.05.

## Results

### Salt Tolerance Assay of Cotton Cultivars During Germination and Post-Germination Stage

**FIGURE 1:**
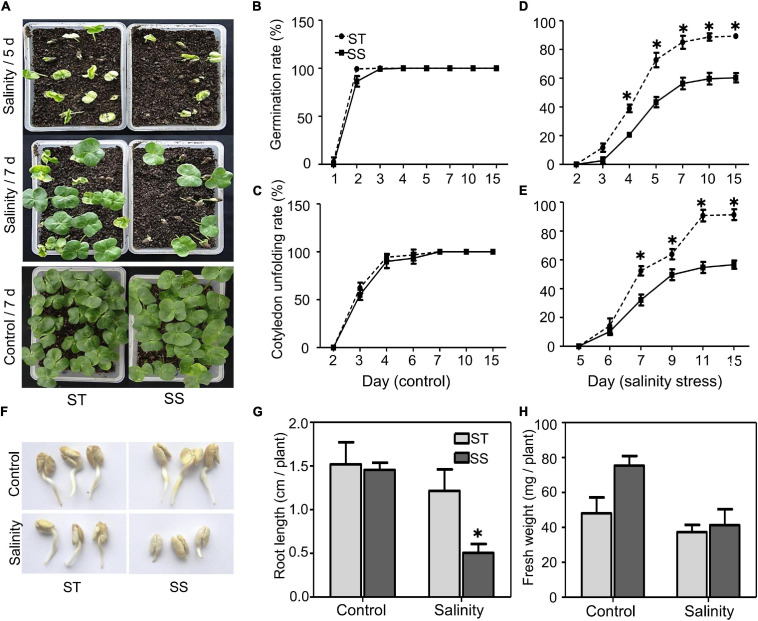
Analysis of salinity tolerance during germination and post-germination. **(A)** Seed germination under control (water) or salinity stress (100 mM NaCl) conditions were analyzed at the indicated time. **(B–E)** Seed germination (%) and cotyledon unfolding rate (%) were analyzed. Germinated seeds were those that visibly emerged from soil. **(F)** Growth of primary roots under control (water) or salinity stress (100 mM NaCl) were photographed after 2 days treatment. **(G)** Root length and **(H)** fresh weight under control or salinity stress conditions (100 mM NaCl) were measured after 2 days treatment. The data are means ± SD from 25 seeds. The asterisk (*) indicate significant differences at *P* < 0.05 (*t*-test). All experiments were performed in triplicate.

To understand salinity tolerance mechanisms of upland cotton, we identified LMY37 (ST) and ZM12 (SS) as the most salt-tolerant and salt-sensitive cultivars out of 12 cultivars with distinct salinity tolerance based on our field assay (Supplementary Table 1). Our phenotypical assay of ST and SS during germination and postgermination revealed that these two cultivars displayed similar germination and cotyledon-unfolding rates under normal conditions (Figures 1A–C). When exposed to salinity stress, ST performed better than SS plants; about 73% of ST seeds germinated after salinity treatment for 5 days, compared with about 43% in SS. Similarly, ST had nearly twice the cotyledon-unfolding rate as SS after treatment for 7 days (Figures 1D,E).

During postgermination, we did not observe significant between-cultivar differences under normal conditions. When treated with 100 mM NaCl for 2 days, the root elongation and biomass of ST were not significantly affected after salinity stress; however, SS plants exhibited significant inhibition in root growth (Figures 1F–H).

### Transcriptome Profile in Response to Salinity Stress

To obtain a global transcriptome profile of cotton seedlings in response to salinity stress, we performed RNA-Seq on ST and SS plants subjected to salinity stress experiments, producing 12 RNA libraries. The libraries yielded approximately 0.8 billion raw reads with an average GC content of 43.9%. Clean reads were aligned to the reference genome (Yuan et al., 2019), in which 94.29–96.13% reads were mapped to the reference genome, producing 85.76–88.03% of the uniquely mapped reads to the reference genome (Supplementary Table 2).

**FIGURE 2:**
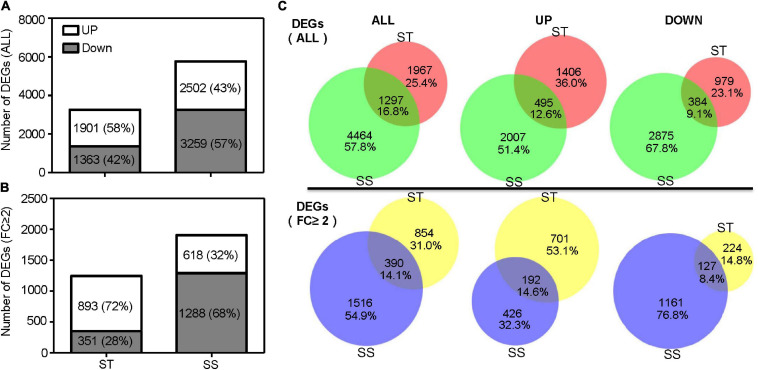
Number of differentially expressed genes (DEGs). **(A)** Histograms show total DEGs and **(B)** DEGs with a 2-fold or greater change in expression. **(C)** Venn diagram illustrates the number of overlapping DEGs between salt-tolerant (ST) and salt-sensitive (SS).

A total of 3,264 DEGs was identified in ST samples, with 1,901 (58%) upregulated and 1,363 (42%) downregulated genes. The SS samples contained 5,761 DEGs, with 2,502 (43%) and 3,259 (57%) up and down regulated genes, respectively (Figure 2A). When considering the DEGs with a 2-fold or greater change in expression (FC ≥ 2), ST had up to 72% upregulated DEGs (893 out of 1,244), more than twice as much as SS (Figure 2B). Of all DEGs, 16.8% (1,297 DEGs) were shared between ST and SS; 25.4% (1,967 DEGs), and 57.8% (4,464 DEGs) specifically responded to salinity stress in ST and SS, respectively (Figure 2C).

### Gene Ontology and KEGG Pathway Analysis

Gene ontology analysis was performed on DEGs from both genotypes; significantly enriched GO terms (padj ≤ 0.05) were categorized into 71 categories, including 37 BP, 5 CC, and 29 MF (Supplementary Table 3). Genes involved in categories related to ROS response were strongly regulated, including “response to oxidative stress,” “oxidoreductase activity,” “peroxidase activity,” and “antioxidant activity.” Categories related to carbohydrate metabolism, such as “cellular carbohydrate metabolic process,” “polysaccharide metabolic process,” and “disaccharide metabolic process,” were also enriched. Other enriched processes include those functioning in cell wall reconstruction, such as “microtubule-based movement,” “microtubule motor activity,” and “microtubule binding” categories (related DEGs are listed in Supplementary Table 4). These results indicated that salinity stress caused extensive changes in metabolic response.

**FIGURE 3:**
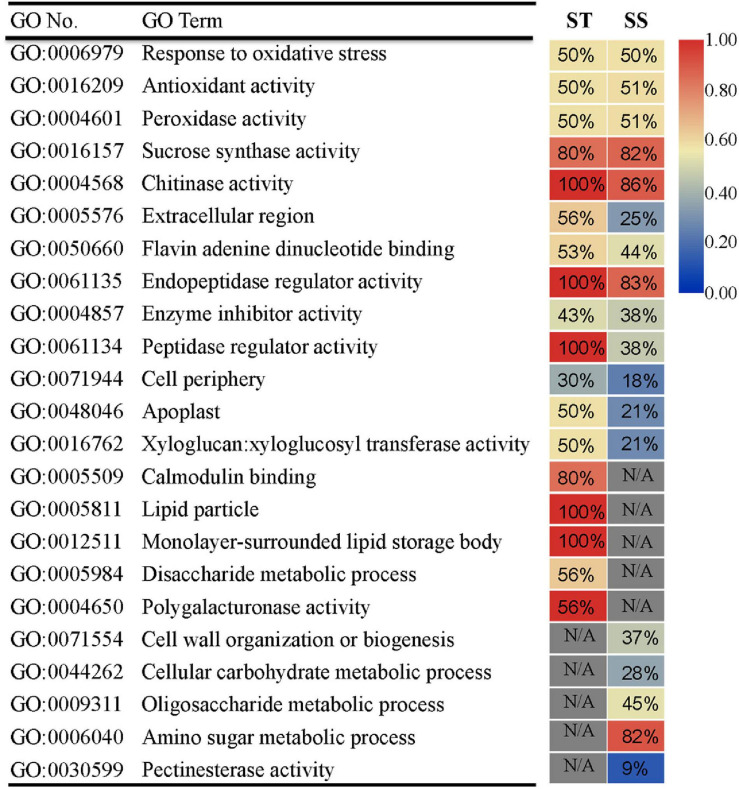
Gene ontology (GO) enrichment analysis of DEGs (FC ≥ 2) in ST or SS. The heat map indicates the ratio of upregulated genes for each GO term. N/A indicates absence of DEGs in enriched GO terms. Only the top 18 most enriched GO categories of each genotype are shown.

To assess the more prominent differences in transcriptional response between ST and SS, we performed GO analysis on DEGs exhibiting a relatively strong response (FC ≥ 2) for each genotype. We also dissected DEG expression patterns for each GO term. The two cultivars had commonly enriched GO terms and unique ones. Thirteen GO terms were shared between ST and SS (e.g., “response to oxidative stress” and “sucrose synthase activity”), indicating that these biological processes may be basic responses to salinity stress across genotypes (Figure 3). Notably, some common GO terms had different DEG expression trends. For example, as seen in ‘peptidase regulatory activity’ term, 100% of DEGs in ST were upregulated, but only 38% was upregulated in SS. Specifically enriched GO terms included calcium-signaling-related “calmodulin binding” in ST and “cell wall organization or biogenesis” in SS. The DEGs in these unique GO terms and those with varying expression patterns in shared GO terms may be responsible for salinity-tolerance variability between genotypes.

Our KEGG pathway analysis revealed two shared pathways (phenylpropanoid biosynthesis and valine, leucine, and isoleucine degradation pathways) between the ST and SS phenotypes. Most related DEGs were downregulated in both cultivars. Other significantly enriched pathways showed genotype specificity, with the majority of involved genes being upregulated. Examples of specifically enriched pathways for ST include the MAPK signaling pathway, which is key in ROS signaling, and the carotenoid biosynthesis pathway to produce an ABA precursor. In SS, a specifically enriched pathway is cysteine and methionine metabolism, which produces the precursor of ET (Table 1).

**TABLE 1:** Kyoto encyclopedia of genes and genomes (KEGG) analysis of differentially expressed genes (DEGs) from salt-tolerant (ST), and salt-sensitive (SS) samples.

**KEGG ID**	**Description**	**DEGs**			***P*-value**
		**Up**	**Down**	**Count**	
**ST (ALL)**					
ath04016	MAPK signaling pathway-plant	8	3	11	7.47E-04
ath00940	Phenylpropanoid biosynthesis	3	8	11	4.00E-04
ath00196	Photosynthesis-antenna proteins	9	0	9	4.91E-09
ath00280	Valine, leucine and isoleucine degradation	2	6	8	1.41E-04
ath00710	Carbon fixation in photosynthetic organisms	6	2	8	8.39E-04
ath00071	Fatty acid degradation	2	4	6	1.37E-03
**SS (ALL)**					
ath00940	Phenylpropanoid biosynthesis	6	13	19	1.43E-05
ath00270	Cysteine and methionine metabolism	10	9	19	2.80E-06
ath00564	Glycerophospholipid metabolism	3	10	13	2.58E-03
ath00920	Sulfur metabolism	8	3	11	2.76E-06
ath00053	Ascorbate and aldarate metabolism	6	3	9	1.25E-03
ath00280	Valine, leucine and isoleucine degradation	0	9	9	2.82E-03
ath00565	Ether lipid metabolism	0	7	7	4.08E-04
**ST (FC ≥ 2)**					
ath00940	Phenylpropanoid biosynthesis	2	6	8	2.01E-06
ath04016	MAPK signaling pathway-plant	5	0	5	2.90E-03
ath00906	Carotenoid biosynthesis	3	0	3	1.92E-03
**SS (FC ≥ 2)**					
ath00940	Phenylpropanoid biosynthesis	2	9	11	1.18E-07
ath00564	Glycerophospholipid metabolism	2	4	6	2.23E-04

**FIGURE 4:**
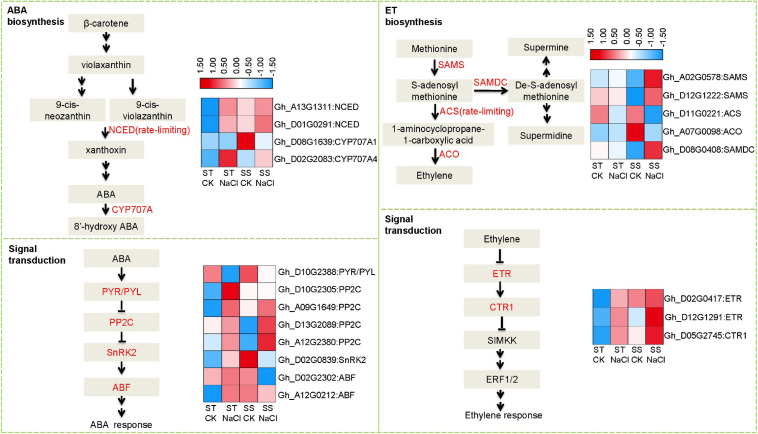
Differentially expressed genes involved in abscisic acid (ABA) and ethylene (ET) biosynthesis and signal transduction. Fragments Per Kilobase of transcript sequence per Millions base pairs sequenced (FPKM) values of related DEGs represented as a color gradient from low (blue) to high (red).

Because stress hormones like ABA and ET play important roles in response to salinity stress, we further analyzed expression patterns of DEGs involved in their biosynthesis and signaling. Several rate-limiting synthetases and key elements showed altered expression, including those that regulate ABA homeostasis: 9-*cis*-epoxycarotenoid dioxygenase (NCED) and cytochrome p450 family 707 (CYP707A) (Figure 4). Both genes were obviously upregulated after salinity stress in ST samples, but in SS, no significant difference was observed other than CYP707A1 showing down-regulation. Accordingly, we observed dramatic alterations in downstream signal transduction elements, such as ABA multiple receptors pyrabactin resistance1/PYR1-like (PYR/PYL) and negative regulator protein phosphatases 2C (PP2C). *s*-adenosylmethionine synthase (SAMS), 1-aminocyclopropane-1-carboxylate synthase (ACS), 1-aminocyclopropane-1-carboxylate oxidase 1 (ACO), *s*-adenosylmethionine decarboxylase proenzyme (SAMDC), and ACS as the rate-limiting factor, are Key enzymes in ET biosynthesis. Under salinity stress, ACS and ACO expressions were depressed in SS, while SAMDC and SAMS were activated. In contrast, these genes did not change in expression in ST after salinity-stressing, although some elements in ET signal transduction pathways showed altered expression, including ethylene receptor (ETR), a negative regulator of the pathway. These data suggest that salinity stress affected the homeostasis of stress hormones in both salt-sensitive and salt-tolerant varieties, leading to different signal transduction and adaption responses.

### Transcription Factors and ROS-Related DEGs in Response to Salinity

**FIGURE 5:**
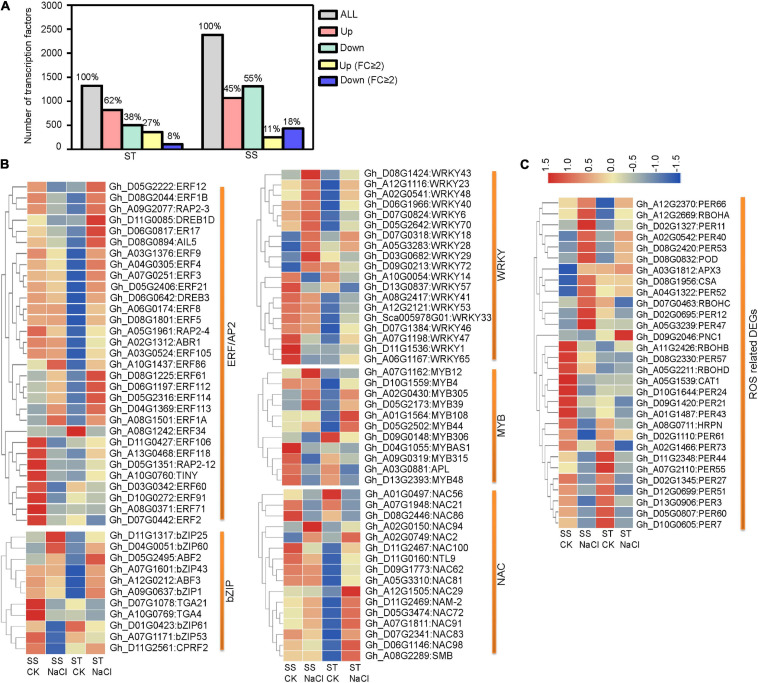
Differentially expressed transcription factor- (TF) and reactive oxygen species- (ROS) related genes in ST and SS were analyzed. **(A)** Histograms show all TF genes and DEGs with a 2-fold or greater change in expression. **(B)** Heatmaps show FPKM values of five TF DEGs and **(C)** ROS-related DEGs in ST and SS. The FPKM values are represented with a color gradient from low (blue) to high (red).

Transcription factors (TFs) are important regulators of stress tolerance and critical downstream regulatory components of the MAPK stress-response cascade. In our data, more TF genes were upregulated in ST than in SS. This pattern became more distinct when considering strongly responsive DEGs (FC ≥ 2) (Figure 5A). Our analysis of five stress-tolerance TF families revealed that ERF/AP2s TFs, key regulators in ET signaling, were the largest group (31 DEGs), followed by WRKYs (19 DEGs), NACs (16 DEGs), bZIPs (11 DGEs), and MYBs (11 DGEs) (Figure 5B). For hormone-response-related ERF/AP2 TFs, 67.74% (21 out of 31) showed strong responses (FC ≥ 2) to salinity stress. In addition, 18 ERF genes showed contrary expression patterns between ST and SS, including *Gh_D05G2222* (*ERF12*), *Gh_D11G0085* (*DREB1D*), and *Gh_D08G2044* (*ERF1B*). In the other four TF families, fewer members were differentially expressed across cultivars (Supplementary Table 5). These various TF genes may regulate the expression of downstream target genes under salinity stress.

Preventing ROS from reaching harmful levels is one of the most important protective mechanisms in stressed plant cells. We identified a number of DEGs related to ROS hemostasis (Figure 5C and Supplementary Table 5). One notable example is the *Rboh* family, responsible for mediating ROS generation under stress conditions. The expression of *RbohD* (*Gh_A05G2211*), whose homolog in *Arabidopsis* is required for salinity-induced antioxidant defense (Ma et al., 2012), is obviously downregulated in SS but slightly upregulated in ST samples. Additionally, several key ROS scavenger-encoding genes, such as *CAT1* (*Gh_A05G1539*) and *PNC1* (*Gh_D09G2046*), were inhibited in SS, but upregulated in ST.

### qRT-PCR Validation of RNA-Seq Data

To validate the RNA-seq data, we use qRT-PCR to check the expressions of 12 genes, such as DEGs involved in phytohormone biosynthesis and signaling, ROS homeostasis as well as TFs genes (Supplementary Table 6 and Supplementary Figure 1). The expression patterns of all these genes from qRT-PCR are consistent with the DEGs data from RNA-seq analysis, suggesting that the RNA-seq data are reliable and reproducible.

### *GhERF12* Function in Salinity Tolerance

**FIGURE 6:**
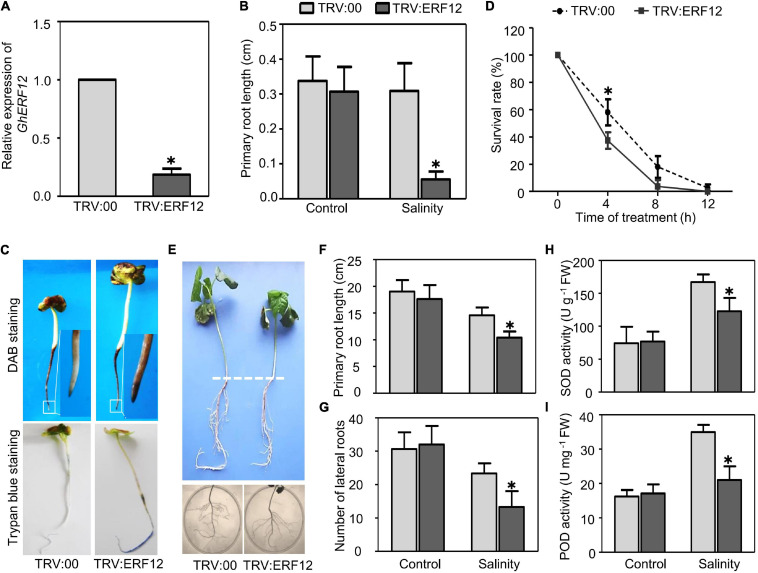
Silencing of *GhERF12* in cotton increased sensitivity to salinity. **(A)** Relative abundance of *GhERF12* transcripts in *GhERF12*-silenced (TRV:ERF12) and control (TRV:00) plants were analyzed with qRT-PCR after 10 days inoculation. *GhActin9* was used as an internal control. Data represent means ± SD (*n* = 6) from three independent replicates. **(B)** Length of primary roots in *GhERF12*-silenced and control plants treated with 100 mM NaCl were measured at the indicated time. **(C)** DAB and Trypan blue stain analysis were used to quantify ROS accumulation and cell death after salinity stress. **(D)** Survival rate of roots from *GhERF12*-silenced and control plants. **(E)** *GhERF12*-silenced and control adult plants (30-day old) treated with 250 mM NaCl for 7 days. **(F)** Root length and **(G)** number of lateral roots in *GhERF12*-silenced and control adult plants. **(H)** Activity of SOD and **(I)** POD in *GhERF12*-silenced and control plants after 250 mM NaCl treatment for 8 h. The data are means ± SD (*n* = 10). The asterisk (^∗^) indicate significant differences at *P* < 0.05 (*t*-test). All experiments were performed in triplicate.

The AP2/ERF TF family genes showed the strongest response to salinity based on the number of TF DEGs and changes to gene expression. For further functional analysis, we selected one representative DEG between ST and SS (upregulated over 4-fold in ST, downregulated around 4-fold in SS) as a candidate salinity-tolerance gene, the ERF/AP2 family member *GhERF12* (*Gh_D05G2222*). First, SSA–VIGS method was used to silence *GhERF12* in very young seedlings and roots. Approximately 8–10 days after germination and inoculation, the roots of pTRV:ERF12 (*GhERF12* silenced) and pTRV:00 (empty vector control) plants were collected to detect *GhERF12* transcripts by qRT-PCR (primers were listed in Supplementary Table 6). *GhERF12* silenced plants had nearly 18% fewer transcripts than control seedlings under normal conditions (Figure 6A). The pTRV:ERF12 and pTRV:00 plants were then exposed to 100 mM NaCl to assess their salt tolerance. The length of primary roots from *GhERF12* silenced plants were significantly depressed compared to the vector control under salinity stress (100 mM NaCl for 12 h) (Figure 6B). Additionally, higher ROS accumulation and more dead cells were detected in pTRV:*GhERF12* roots by DAB and Trypan blue staining, respectively (Figures 6C,D). Meanwhile, no significant differences were observed between silenced and vector control seedlings under normal conditions (Supplementary Figure 2). Furthermore, a stronger stress treatment (250 mM NaCl for 7 days) on adult (30-day-old) plants with true leaves and complete root systems were carried out. The pTRV:*GhERF12* plants showed significantly shorter primary roots and fewer lateral roots than control plants (Figures 6E–G). We also found out that *GhEFR12* silenced plants exhibited greater inhibition of ROS-scavenging enzymes (including POD and SOD) under stress conditions than control, despite no obvious differences under normal conditions (Figures 6H,I). These results suggest that *GhERF12* is involved in salinity tolerance through influencing ROS levels, specifically via regulating antioxidant enzyme activities under stress conditions.

**FIGURE 7:**
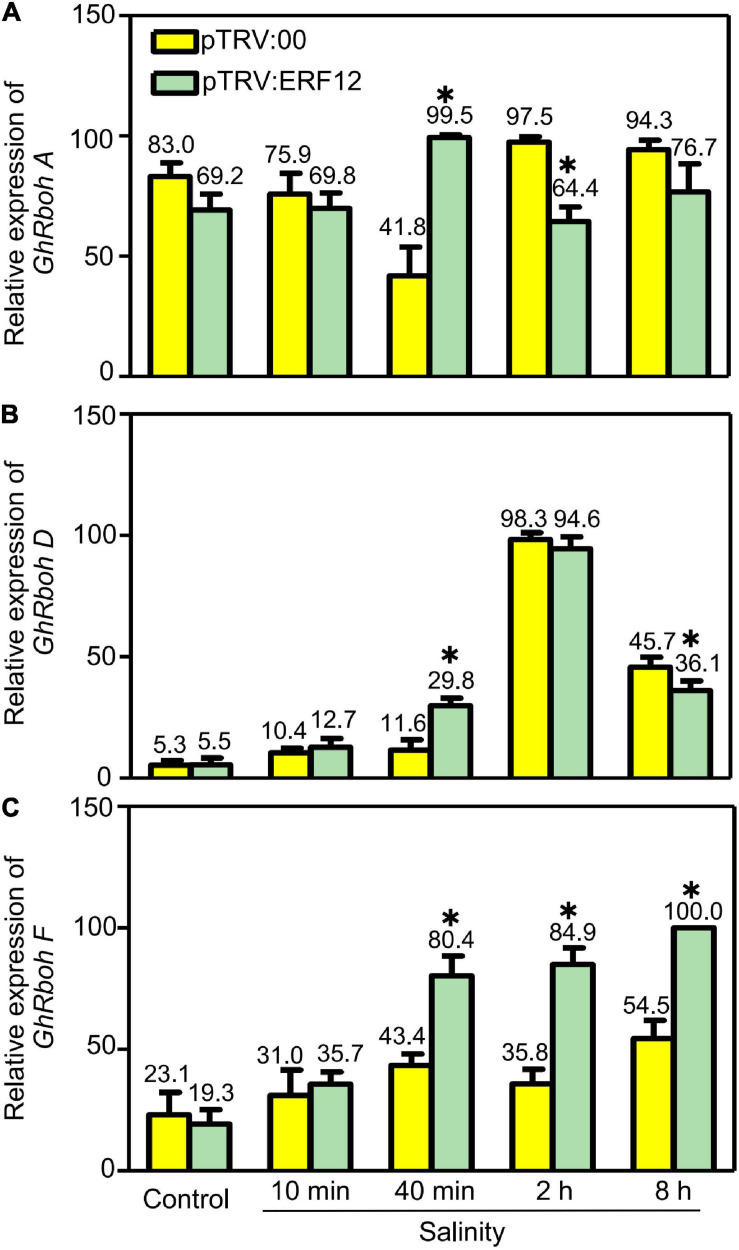
Relative abundance of *GhRboh* genes (*GhRboh A, D, and F*) in *GhERF12*-silenced (TRV:ERF12) and control (TRV:00) plants were determined with qRT-PCR at the given time points. *GhActin9* was used as an internal control. Data represent means ± SD (*n* = 6) from three independent replicates. The asterisk (*) indicate significant differences at *P* < 0.05 (*t*-test).

Reactive oxygen species generation is also important to ROS homeostasis; we checked the expression of respiratory burst oxidase homolog genes, including *GhRbohA* (*Gh_A02G1791*), *GhRboh D* (*Gh_A05G2211*) and *GhRboh F* (*Gh_A12G2653*), for their homologs have been reported to participate in abiotic stress (salinity or drought) induced ROS production in other plants (Ma et al., 2012; Wang et al., 2016). During short stress treatment (40 min), the expression of tested genes was significantly enhanced in *GhERF12-*silenced plants when compared to the vector control. When treated for 2 or 8 h, the expression of *GhRboh A* and *GhRboh D* were significantly depressed; while, the expression of *GhRboh F* enhanced in *GhERF12*-silenced plants compared to the vector control (Figure 7). These results indicated that *GhERF12* could affect the expression of *GhRboh A, D*, and *F* at transcription level under stress conditions.

## Discussion

Germination and post-germination stages are the basis of propagation, making them critical periods in plant growth and development. Seedlings at this stage usually exhibit higher sensitivity to environmental stress. Here, we successfully analyzed phenotypic and transcriptomic differences between salt-tolerant and salt-sensitive cotton cultivars. Our findings expand current knowledge of plant salinity tolerance and provide useful genes for improving tolerance during early development.

Transcriptomic analysis is widely used to explore stress-tolerant genes and related mechanisms in plants (Orsini et al., 2010; Peng et al., 2014, 2018; Li et al., 2016). We identified a large number of salinity-responsive genes, and their expression patterns clearly differed between genotypes. We found more DEGs in SS, with the majority downregulated, whereas ST had fewer DEGs and most were upregulated (Figure 2). These results indicated that the tolerant genotype needs fewer genes to mitigate salt-induced damage and more genes were activated in response to survival under stress conditions. Our conclusion is consistent with previous studies in rice (Walia et al., 2005) and eggplant (Li J. et al., 2019).

Antioxidants are one of the most important protective mechanisms against salinity stress in plants. In line with this general consensus and indicative of antioxidants being a general salt-tolerance mechanism during early development, we found numerous genes associated with “response to oxidative stress,” “antioxidant activity,” and “peroxidase activity” that were expressed in both varieties. Cell-wall modification also mediates plant acclimatization to salinity stress. In a study on salt-tolerant and -sensitive soybean genotypes, pectin increase in the cell wall was found to be beneficial for root growth under salinity stress (An et al., 2014). In addition, genes related to cell wall remodeling are involved in salinity tolerance. For example, salinity tolerance in *Arabidopsis* increased with overexpression of *RhEXPA4*, a key gene involved in cell-wall loosening (Lu et al., 2013). For both ST and SS, our study found enrichment of GO terms and KEGG pathways associated with cell wall modulation, including “cell wall,” “microtubule-based process,” “xyloglucan,” “chitinase activity,” and “phenylpropanoid biosynthesis.” Thus, our findings corresponded to previous research.

We observed obvious between-genotype differences in signaling and response events. Specifically, ABA and ET hormones were dramatically altered under salt stress. ABA mediates responses to salinity mainly through modulating metabolism and downstream target genes during signal transduction (Shi et al., 2013; Xie et al., 2016). As key regulators of ABA biosynthesis, NCED1 and CYP707A play critical roles in influencing ABA accumulation (Kim et al., 2009). Other important regulators of stress tolerance include ABA downstream targets, such as ABA receptor PYR/PYL, regulator PP2C, and ABA response factor ABF. Similarly, key components in ET signal transduction also participate in salinity tolerance. There are five ET receptors: ETR1, ETR2, ERS1 (ethylene response sensor 1), ERS2, and EIN4 (Wang et al., 2002). Loss-of-function mutant *etr1* is unresponsive to ET and was more salt-sensitive than wild type (Wang et al., 2008). Additionally, constitutive triple response 1 (CTR1) is a protein kinase that negatively regulates ET signaling and is involved in salt stress response. Silencing *ctr1-1* in *Arabidopsis* caused a constitutive ET reaction and strong salt tolerance during seed germination and development (Achard et al., 2006). In our data, ST and SS differentially expressed these key elements related to ABA and ET biosynthesis and signaling. We also identified more DEGs in ABA metabolism and signaling, and found stronger changes to the expression of linked genes in ST than in SS (Figure 4).

Furthermore, ERFs are another group of molecules that regulate stress tolerance. They are characterized by a conserved AP2/ERF motif and by interacting with the promoter elements containing the GCC box (AGCCGCC) or DRE motif (CCGAC) (Phukan et al., 2017). Our data revealed several TF genes related to salt-stress adaptation, with ERF/AP2 TF genes exhibiting a stronger response to salinity than other TFs (Figure 5 and Supplementary Table 5). We propose that these responsive genes may regulate target genes during stress response. Indeed, some of our identified ERF/AP2 genes were previously reported as involved in stress tolerance, such as ERF5 (Pan et al., 2012; Gharbi et al., 2017; Illgen et al., 2020) and DREB1D (Guttikonda et al., 2014; Alves et al., 2017). Taken together, stronger ABA and ET responses appear to be factors in higher salinity tolerance.

**FIGURE 8:**
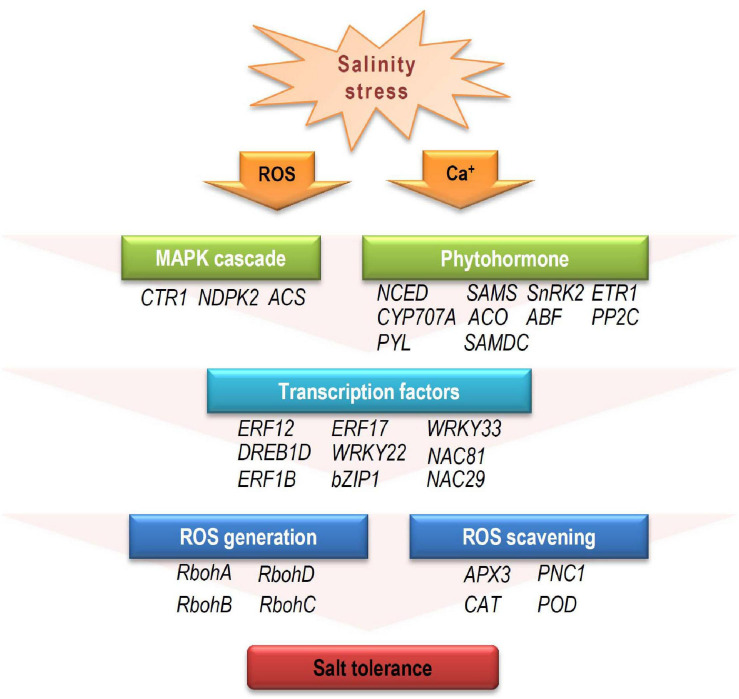
The potential response model to salinity stress in cotton during early developmental stage. When exposed to salinity stress, the resultant stress signal alters secondary messengers, such as calcium (Ca^2+^), ROS, and stress hormones (ABA and ET). Downstream MAPK cascades influence expression of TFs (e.g., AP2/ERF, MYB, NAC, WRKY, and bZIP) that regulate target gene expression. These target genes are involved in ROS generation and scavenging, leading to varying salinity-stress responses. CTR1, constitutive triple response 1; NDPK2, nucleoside diphosphate kinase 2; ACS, 1-aminocyclopropane-1-carboxylic acid synthase; NCED, 9-*cis*-epoxycarotenoid dioxygenase; SAMS, s-adenosylmethionine synthase; SnRK2, sucrose nonfermenting 1 (SNF1)-related protein kinase 2; ETR1, ethylene response 1; CYP707A, cytochrome p450 family 707A; ACO, 1-aminocyclopropane-1-carboxylate oxidase 1; ABF, ABRE-binding factor; PP2C, protein phosphatases 2C; PYL, pyrabactin resistance1/PYR1-likes; SAMDC, s-adenosylmethionine decarboxylase proenzyme; RbohA-D, respiratory burst oxidase homologs A-D; APX3, ascorbate peroxidase 3; PNC1, cationic peroxidase 1; CAT, catalase; POD, peroxidase.

Based on our analysis of the RNA-seq data, a model for the mechanism during salinity stress at early developmental stage was proposed. When young seedlings were exposed to salinity stress, the external receptors firstly receive stress signal, and secondary messengers (e.g., ROS, ABA, and ET) activate the downstream elements, including MAPK cascades, amplifying the stress signals. For the stress hormone messengers (ABA and ET), to maintain their contents at a proper level and conduct the signal is important under stress conditions (Wilkinson and Davies, 2010; Leng et al., 2014; Pan et al., 2019). A number of genes involved in hormone biosynthesis and signal transduction participate in this process, such as *NCED*, *PYL*, and *ACO*. Then, these proteins affect their downstream TFs (such as ERFs, WRKYs, NACs, and bZIPs), which regulate the expression patterns of targeted functional genes involved in ROS generation and scavenging at the transcriptional level. These differential expression of signaling elements and downstream functional genes may directly or indirectly affect salinity tolerances (Figure 8).

Finally, *GhERF12* was selected for functional analysis for its strong responsive to salinity stress, with its expression increased in ST and dramatically downregulated in SS. It has been reported that ERF12 is a member of ERF subfamily B-1 which suppress DRE-mediated transcription of cold- or drought-inducible genes (Ohta et al., 2001). In addition, it is a key seed-dormancy regulator in an ETR1/RDO3-mediated downstream pathway of ET signaling (Li X. et al., 2019). Here, we observed that salinity strongly upregulated *GhERF12* expression in ST, and the silencing of *GhERF12* enhanced sensitivity to salinity. These results indicate that *GhERF12* may play a critical role in salinity tolerance during post germination. Previous studies have shown that ERF/AP2 TFs usually modifies ROS scavenging to regulate stress tolerance. Research in *Tamarix hispida* found that ERF subfamily member ThCRF1 improved salt tolerance via enhancing trehalose and proline biosynthesis, as well as improving SOD and POD activities to increase ROS scavenging capability (Qin et al., 2017). A recent study in tobacco revealed that ERF172 could directly bind to the promoter region of the *NtCAT* gene, positively regulating its expression and improving drought-stress tolerance; the mechanism partially involves CAT regulation to mediate H_2_O_2_ homeostasis (Zhao et al., 2020). Our results here were similar: POD and SOD activity experienced a greater decline after silencing *GhERF12*, indicating that *GhERF12* may contribute to salinity-stress response through regulating ROS scavenging. On the other hand, we screened the expression of *GhRboh* genes in *GhERF12*-silenced plants after salinity stress. It turned out that silencing of *GhERF12* caused upregulation of *GhRbohA*, *GhRbohD*, and *GhRbohF* in gene silenced plants, indicating that *GhERF12* may participate in stress induced ROS generation.

## Conclusion

In this study, we performed the comparative phenotypic and transciptomic analysis of two cotton cultivars in response to salt stress during early developmental stage. GO and KEGG analysis demonstrated that DEGs belonging to a wide range of biological processes are involved in salinity tolerance response. The different expressions of genes involved in ET and ABA biosynthesis and signaling, as well as the distinct expression of downstream TF genes are predicted to be closely linked to salt tolerance. Moreover, a model for the mechanism during salinity stress at early developmental stage was proposed, expanding the understanding of plant salinity tolerance. Finally, *GhERF12* was functionally analyzed and turned out to be an important regulator by affecting ROS balance under salinity stress. Our data extend the view on molecular response to salt stress and supply candidate genes for salt-tolerant breeding in cotton.

## Data Availability Statement

The data presented in the study are deposited in the NCBI SRA BioProject repository, and the accession number is PRJNA684671.

## Author Contributions

JuZ received grant support. JuZ and FW designed the experiments, provided cotton cultivar/germplasm, and revised the manuscript. JiZ and PZ performed salt tolerance analysis, RNA samples preparation, transcriptomic analysis, gene function analysis, and prepared the manuscript. XH, YG, YC, and ZS participated in the field experiments, data collection, and analysis. All authors read and approved the final manuscript.

## Conflict of Interest

The authors declare that the research was conducted in the absence of any commercial or financial relationships that could be construed as a potential conflict of interest.

## Footnotes

1 http://imagej.nih.gov/ij

2 http://www.genome.jp/kegg/

3 http://www.invitrogen.com

## Supplementary Material

Supplementary Figure 1 Validation of RNA-seq data by qRT-PCR.

Supplementary Figure 2 Phenotypes of pTRV:ERF12 and pTRV:00 plants under normal conditions.

Supplementary Table 1 Salt tolerance analysis of different cotton cultivars.

Supplementary Table 2 Quality assessment of raw RNA-seq data.

Supplementary Table 3 Gene ontology (GO) analysis of DEGs from ST and SS.

Supplementary Table 4 Differentially expressed genes (DEGs) involving in enriched GO terms.

Supplementary Table 5 Expression of TF and ROS related genes.

Supplementary Table 6 Primers in this study.
