# Supplementary figures and images for: Comparative Phenotypic and Transcriptomic Analysis Reveals Key Responses of Upland Cotton to Salinity Stress During Postgermination
Source: Front Plant Sci. 2021 Apr 13;12:639104. doi: 10.3389/fpls.2021.639104 (PMC8076740; doi:10.3389/fpls.2021.639104)

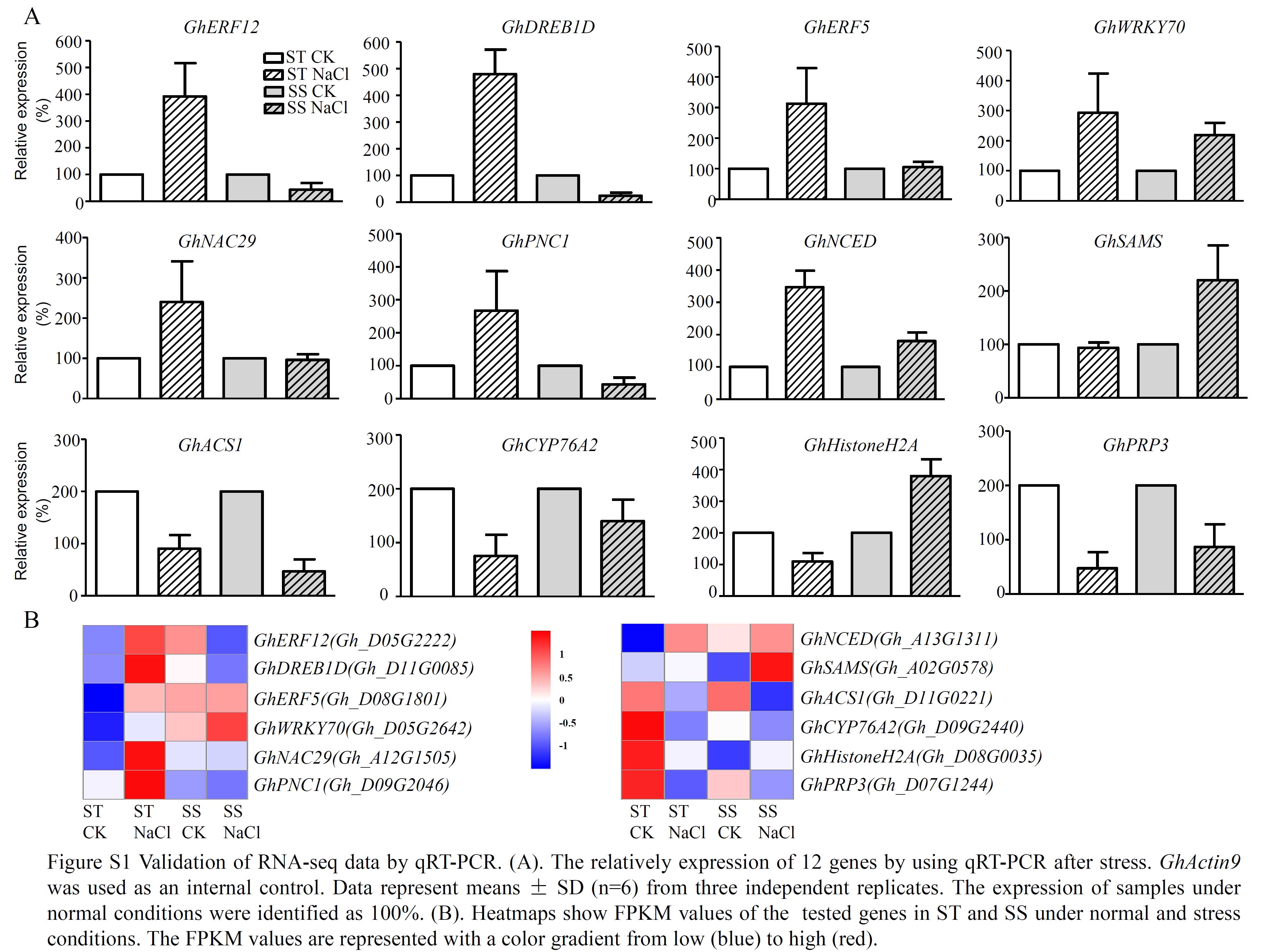

Supplement: Supplementary Figure 1 — Validation of RNA-seq data by qRT-PCR. [file Image_1.jpg]

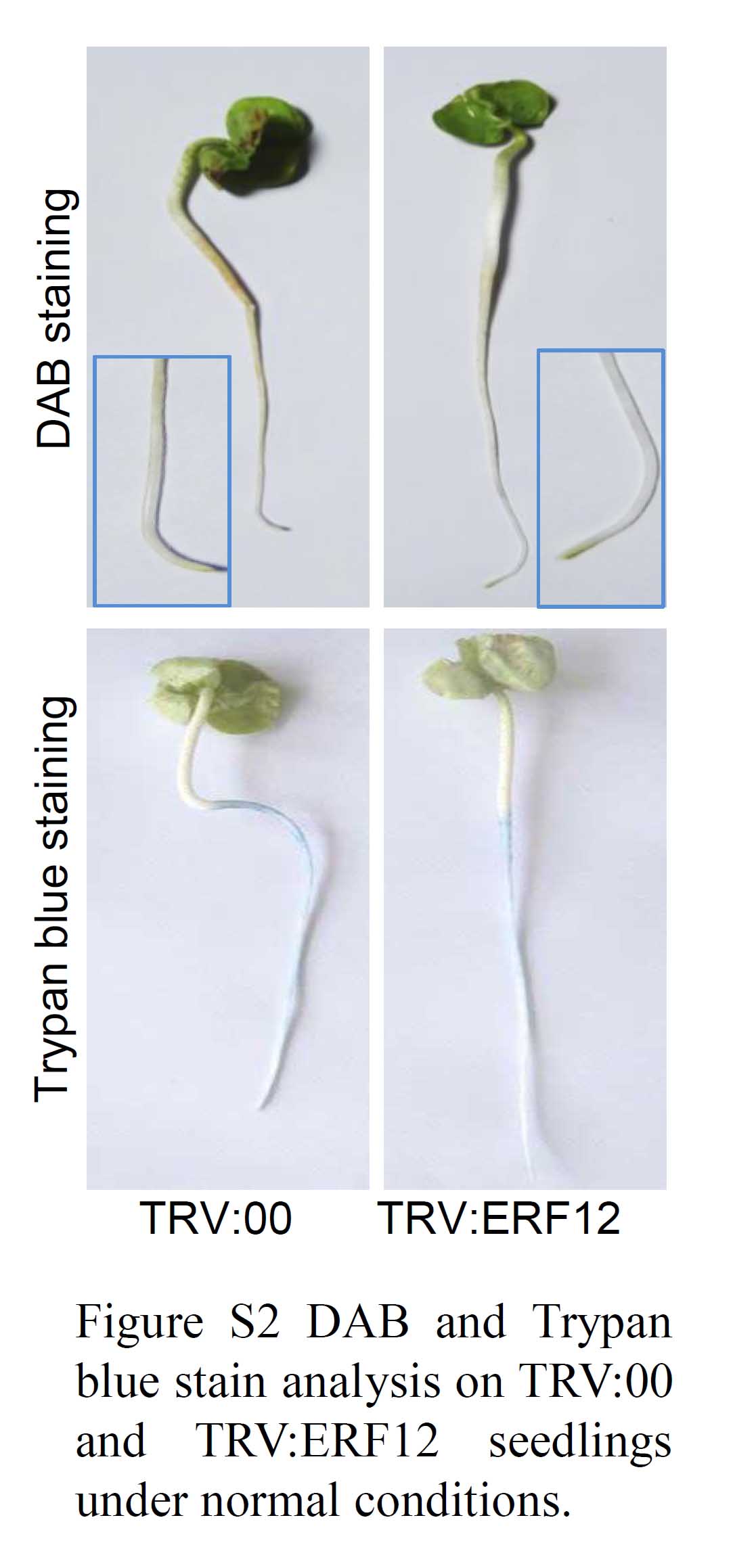

Supplement: Supplementary Figure 2 — Phenotypes of pTRV:ERF12 and pTRV:00 plants under normal conditions. [file Image_2.jpg]
