# Supplementary material for: Comparative Phenotypic and Transcriptomic Analysis Reveals Key Responses of Upland Cotton to Salinity Stress During Postgermination
Source: Front Plant Sci. 2021 Apr 13;12:639104. doi: 10.3389/fpls.2021.639104 (PMC8076740; doi:10.3389/fpls.2021.639104)
Supplement: Supplementary Table 1 — Salt tolerance analysis of different cotton cultivars. [file Table_1.docx]

**TABLE S1 Germination and Survival Rate of 12 Different Cotton Varieties after Salinity Stress**

|  | Code | Germination rate（%） | | |  | Survival rate（%） | | |
| --- | --- | --- | --- | --- | --- | --- | --- | --- |
|  |  | 0.40% | 0.60% | 0.80% |  | 0.40% | 0.60% | 0.80% |

| **1** | LMY37 | 100±0.58a | 99±3.28a | 83±1.53a |  | 100±0.00a | 99.8±1.88a | 99.0±1.88a |
| --- | --- | --- | --- | --- | --- | --- | --- | --- |

| **2** | Z9807 | 97±2.31ab | 91±6.11ab | 68±5.69b |  | 100±0.00a | 99.0±1.67a | | 96.5±1.90ab |
| --- | --- | --- | --- | --- | --- | --- | --- | --- | --- |
| **3** | J24 | 94±3.52ab | 91±5.13b | 70±5.57b |  | 85.7±4.29d | 85.8±4.76e | | 83.2±2.81ef |
| **4** | L7619 | 98±1.53ab | 91±3.22ab | 71±1.79b |  | 92.7±1.21b | 91.3±5.20d | | 90.9±1.86cd |
| **5** | L6 | 94±4.04ab | 89±6.11b | 68±5.25b |  | 87.4±3.82bc | 91.2±3.48cd | | 89.3±2.63abc |
| **6** | L6269 | 95±3.06ab | 85±5.51b | 71±3.51b |  | 91.4±1.67ab | 92.7±1.73bcd | | 92.0±3.58cd |
| **7** | L1 | 91±5.03bc | 85±4.51b | 74±4.04b |  | 96.6±1.33ab | 94.8±1.35ab | | 95.8±5.27ab |
| **8** | H7860 | 86±3.51cd | 76±6.43c | 61±4.61c |  | 88.6±2.97bc | 90.3±3.55bcd | | 89.4±1.61cd |
| **9** | ZZ12 | 83±3.05d | 76±4.04c | 45±4.95d |  | 81.7±3.78cd | 86.3±6.03e | | 83.9±3.408e |
| **10** | Y21 | 68±2.52e | 56±4.51d | 39±2.65de |  | 89.8±4.66bc | 89.9±1.66bc | | 89.8±4.27bc |
| **11** | J1 | 66±6.51ef | 53±3.06de | 37±2.08e |  | 95.1±1.19ab | 92. 0±4.61bc | | 93.5±4.38d |
| **12** | ZM12 | 60±4.51f | 45±3.16e | 23±3.51f |  | 90.1±1.49b | 89.5±3.16e | | 90.2±6.31f |
|  |  |  |  |  |  |  |  |  | |
|  |  |  |  |  |  |  |  |  | |
