# Supplementary material for: Comparative Phenotypic and Transcriptomic Analysis Reveals Key Responses of Upland Cotton to Salinity Stress During Postgermination
Source: Front Plant Sci. 2021 Apr 13;12:639104. doi: 10.3389/fpls.2021.639104 (PMC8076740; doi:10.3389/fpls.2021.639104)
Supplement: Supplementary Table 2 — Quality assessment of raw RNA-seq data. [file Table_2.docx]

**TABLE S2. Quality assessment of raw RNA-seq data**

| Sample | Raw reads | Clean reads | GC  (%) | Total mapped  (%) | Unique mapped  (%) | Multiple mapped  (%) |
| --- | --- | --- | --- | --- | --- | --- |
| ST_CK1 | 64986102 | 64248496 | 43.86 | 96.02 | 87.72 | 8.29 |
| ST_CK2 | 67576382 | 66630996 | 43.81 | 95.83 | 87.18 | 8.65 |
| ST_CK3 | 58584438 | 57374700 | 43.91 | 95.55 | 86.45 | 9.10 |
| ST_NaCl1 | 55995904 | 54910418 | 44.24 | 95.67 | 86.70 | 8.96 |
| ST_NaCl2 | 67751284 | 65577034 | 43.56 | 95.75 | 86.71 | 9.04 |
| ST_NaCl3 | 61810190 | 60486008 | 43.60 | 94.29 | 85.76 | 8.53 |
| SS_CK1 | 69011064 | 68114690 | 43.84 | 95.96 | 87.81 | 8.15 |
| SS_CK2 | 71860804 | 70984260 | 43.88 | 96.13 | 88.02 | 8.11 |
| SS_CK3 | 60244350 | 59566826 | 43.90 | 96.09 | 88.03 | 8.06 |
| SS_NaCl1 | 64046360 | 63153804 | 44.37 | 95.24 | 86.62 | 8.63 |
| SS_NaCl2 | 64951574 | 64216460 | 43.86 | 95.53 | 87.69 | 7.83 |
| SS_NaCl3 | 84221940 | 83150222 | 43.92 | 95.87 | 87.95 | 7.93 |
